# Single Nucleotide Polymorphism–Based Validation of Exonic Splicing Enhancers

**DOI:** 10.1371/journal.pbio.0020268

**Published:** 2004-08-31

**Authors:** William G Fairbrother, Dirk Holste, Christopher B Burge, Phillip A Sharp

**Affiliations:** **1**Center for Cancer Research, Massachusetts Institute of TechnologyCambridge, MassachusettsUnited States of America; **2**Department of Biology, Massachusetts Institute of TechnologyCambridge, MassachusettsUnited States of America; **3**McGovern Institute, Massachusetts Institute of TechnologyCambridge, MassachusettsUnited States of America

## Abstract

Because deleterious alleles arising from mutation are filtered by natural selection, mutations that create such alleles will be underrepresented in the set of common genetic variation existing in a population at any given time. Here, we describe an approach based on this idea called VERIFY (variant elimination reinforces functionality), which can be used to assess the extent of natural selection acting on an oligonucleotide motif or set of motifs predicted to have biological activity. As an application of this approach, we analyzed a set of 238 hexanucleotides previously predicted to have exonic splicing enhancer (ESE) activity in human exons using the relative enhancer and silencer classification by unanimous enrichment (RESCUE)-ESE method. Aligning the single nucleotide polymorphisms (SNPs) from the public human SNP database to the chimpanzee genome allowed inference of the direction of the mutations that created present-day SNPs. Analyzing the set of SNPs that overlap RESCUE-ESE hexamers, we conclude that nearly one-fifth of the mutations that disrupt predicted ESEs have been eliminated by natural selection (odds ratio = 0.82 ± 0.05). This selection is strongest for the predicted ESEs that are located near splice sites. Our results demonstrate a novel approach for quantifying the extent of natural selection acting on candidate functional motifs and also suggest certain features of mutations/SNPs, such as proximity to the splice site and disruption or alteration of predicted ESEs, that should be useful in identifying variants that might cause a biological phenotype.

## Abstract 2

A computational analysis of single nucleotide polymorphisms showed that one-fifth of mutations that disrupt predicted exonic splicing enhancers have been eliminated by natural selection

## Introduction

Exonic splicing enhancers (ESEs) were identified about a decade ago as short oligonucleotide sequences that enhance exon recognition by the splicing machinery (reviewed in Blencowe 2000 and Cartegni et al. 2002). Sequences with ESE activity have been identified in both plants and animals and have been found to occur frequently in constitutively spliced exons as well as alternatively spliced exons (Tian and Kole 1995; Coulter et al. 1997; Liu et al. 1998; Schaal and Maniatis 1999; Fairbrother et al. 2002). ESEs often mediate their effects on splicing through the action of proteins of the SR protein family, which bind to ESEs and recruit components of the core splicing machinery to nearby splice sites (Graveley 2000).

Previously, we reported a computational method called relative enhancer and silencer classification by unanimous enrichment (RESCUE)-ESE which identifies ESEs in human genomic sequences using statistical properties of the oligonucleotide composition and splice site strengths of large datasets of exons and introns (Fairbrother et al. 2002). This method identified a set of 238 hexamers (of the 4,096 possible hexamers) which were predicted to possess ESE activity on the basis that (1) they are significantly enriched in human exons relative to introns and (2) they are significantly more frequent in exons with weak (nonconsensus) splice sites than in exons with strong (consensus) splice sites. Tests of splicing enhancer activity using an in vivo splicing reporter system confirmed ESE activity for a representative sequence from each of ten clusters of RESCUE-ESE hexamers (Fairbrother et al. 2002). The function of this set of hexamers was further confirmed by the observation that ESE activity was reduced significantly in nine out of ten point mutants chosen to eliminate RESCUE-ESE hexamers and the observation that the set of RESCUE-ESE hexamers was also predictive in analyzing a list of published mutations that cause exon skipping in the human hypoxanthine phosphoribosyl transferase gene (Fairbrother et al. 2002).

A variety of other selection-based methods have been used to identify sets of sequences that are capable of functioning as ESEs. These SELEX methods isolate ESEs from a complex pool of random sequence by iteratively selecting and amplifying the fraction of molecules that can function as ESEs in a reporter assay (Tian and Kole 1995; Coulter et al. 1997; Liu et al. 1998; Schaal and Maniatis 1999). These methods have yielded a variety of sequence motifs, and ESE activity of representative sequences has been demonstrated in reporter systems. Often, these motifs have not been refined to a degree where it is possible to reliably design single point mutations that disrupt ESE function (Tian and Kole 1995; Coulter et al. 1997; Liu et al. 1998; Schaal and Maniatis 1999).

Despite this, a few previous studies have identified several disease alleles where the disruption of a conserved splicing enhancer corresponds to observed splicing defects, a noteworthy example being splicing mutations in the breast cancer gene BRCA1 (Liu et al. 2001; Orban and Olah 2001). To date, this type of analysis has been limited to only a few genes. While mutational studies on model splicing substrates have proven an effective means of characterizing individual ESEs, the ability to draw general conclusions about ESE function has been complicated by additional features that vary between substrates. Features such as transcript secondary structure, adjacent negative elements, and the possible contribution of splicing factors associated with the transcription machinery could all modulate ESE activity on a given substrate and prevent any single substrate from serving as a paradigm for all aspects of exon recognition problems. To dilute the contribution of sequence context and to develop general rules for splicing, we have used genomic data to survey the strength of selection on RESCUE-ESE hexamers 2 across several thousand exons.

Here we test the hypothesis that, in addition to protein-coding requirements, human exon sequences are also significantly constrained by the requirement to encode ESEs. We have developed a population genetic approach (variant elimination reinforces functionality [VERIFY]) which exploits the simple principle that, because they are selected against, deleterious alleles will tend to be underrepresented in the pool of sequence variants that are common in a population (Graur and Li 2000). Taking advantage of the huge repository of genetic information represented by the human single nucleotide polymorphism (SNP) database, we determined the ancestral allele for exonic SNPs by comparison to the chimpanzee genome. This information makes it possible to distinguish between SNPs derived from mutations that disrupt predicted ESEs and those derived from mutations that create predicted ESEs, allowing an assessment of the degree of selection to conserve ESEs in human exons. While we have tested a small subset of RESCUE predictions in a functional assay, many sequences proposed to possess ESE activity have not been validated. This work provides additional evidence that RESCUE-ESE hexamers are physiologically important when they occur in human exons and suggests a way to use SNP data to validate oligonucleotide motifs proposed to have biological activity.

## Results/Discussion

To assess the relationship between the locations of common genetic variation in human genes and ESEs, we screened the public SNP database (dbSNP; build 112) for SNPs that mapped to human exons. A set of biallelic reference SNPs was used, excluding entries that (1) mapped to multiple regions in the human genome, (2) mapped to repetitive elements, or (3) were derived from transcript sequence data, e.g., through comparison of expressed sequence tags (ESTs) (see Materials and Methods for details). The remaining SNPs were searched against a large database of human genes containing approximately 121,000 internal exons annotated by aligning available human cDNAs to the assembled genome using the GENOA genome annotation system (see Materials and Methods). This search identified 9,862 SNPs that were localized to an internal exon (aligned perfectly to the genomic sequence over a 33-base segment centered on the polymorphic position).

### ESE Density Is Highest and SNP Density Lowest near Splice Sites

**Figure 1:**
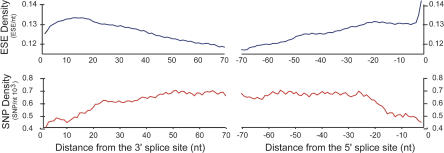
Density of Predicted ESEs and SNPs along Human Exons RESCUE-ESE hexamers were searched against a database of 121,000 internal human exons. ESE density (blue curve) was determined as the fraction of hexamers beginning at the given exon position in this dataset that were contained in the RESCUE-ESE set. SNP density (red curve) was determined analogously using SNPs from dbSNP mapped to the exon database. Both curves were smoothed by averaging the densities over a leading (3′ss) or lagging (5′ss) window of ten nucleotides.

Recording the position of each SNP within the corresponding exon revealed that SNP density is not uniform along exons (Figure 1). Consistent with previous observations (Majewski and Ott 2002), SNP density was approximately 20–30% lower near both the 3′ splice site (3′ss) and the 5′ splice site (5′ss) of human exons than in the interior of exons, and reached a plateau at about 25–30 bases from the splice sites. The distribution of RESCUE-predicted ESE hexamers along exons had roughly an inverse relationship to the SNP density, with the highest density of ESEs observed near the 5′ and 3′ splice junctions and a lower density in the interior of exons (Figure 1). Previously, ESE activity has been observed to vary as a function of the distance between the ESE and the adjacent splice sites, with the highest activity in vitro and in vivo for ESEs positioned closest to splice sites (Nelson and Green 1988; Lavigueur et al. 1993; Graveley et al. 1998). Thus, selective pressure is likely to be higher on ESEs located near splice junctions relative to ESEs in the interior of exons, which could explain the trend in ESE density shown in Figure 1. As a consequence of the increased density of ESEs near splice sites, mutations that occur in exons near splice sites should have a higher likelihood of disrupting ESEs and therefore be more likely to be eliminated by purifying selection. Thus, selection on ESEs could potentially explain the trend in SNP density seen in Figure 1. In order to more directly test the hypothesis that ESE disruption mutations are subject to negative selection, we conducted a large-scale analysis of sequence variation in human exons.

### Estimating the Frequency of ESE Disruption in Human SNPs

The frequency of ESE disruption for simulated (randomly generated, unselected) mutations was compared to the frequency of ESE disruption observed in SNPs, which represent mutations that have survived selection to become reasonably frequent in the human population. SNPs are shaped by the interplay between the mutation process and the process of natural selection. Comparing simulated mutations to natural variations is an effective way to decouple these processes, and this approach has been used by others to study selective pressure in protein-coding genes (Gojobori 1983; Nei and Gojobori 1986; Kowalczuk et al. 2001). Here, we describe the analysis of simulated and natural mutations using a variation of the McDonald–Kreitman test, a widely used statistical test for detecting selection in genes, that we have adapted to measure the strength of selection acting on ESEs using data from several thousand exons (McDonald and Kreitman 1991; Jenkins et al. 1995).

When considering SNPs, in order to distinguish mutations that disrupt predicted ESEs from those that create predicted ESEs, the identity of the ancestral allele must be established. Since the mutations that created most human polymorphisms occurred less than 1 million years ago (Slatkin and Rannala 2000; Miller and Kwok 2001), long after the human–chimpanzee divergence of 5 million years ago (Stauffer et al. 2001), the orthologous chimpanzee exon will almost always represent the sequence of the ancestral allele.

Each of the 9,862 mapped human SNPs described previously was aligned (using a 33-nucleotide sequence window centered around the polymorphic position) to unassembled reads from the genome of the chimpanzee *Pan troglodytes,* accessed through the NCBI trace archives (ftp://ftp.ncbi.nih.gov/pub/TraceDB/pan_troglodytes/). As the trace archives represented several-fold coverage of the chimp genomic sequence, most SNPs matched to several sequence reads. Whenever one allele of a human SNP consistently matched the chimpanzee sequence in all high-quality alignments, that allele was designated as the ancestral allele, and the other allele at that position was designated as a variant allele. The 8,408 SNPs that satisfied this criterion were then annotated for predicted ESEs in both the ancestral and variant sequence, simply by comparing the six overlapping hexanucleotides that differed between the two alleles to the set of RESCUE-ESE hexamers and recording the number of matching hexamers.

It is well known that current SNP databases contain a certain rate of error. The SNP consortium estimated that about five percent of their submissions were false positives attributed to base calling errors (Altshuler et al. 2000). Incorrect mapping can also result in the misclassification of nearly identical paralogous regions as SNPs (Bailey et al. 2002; Cheung et al. 2003). The rate of false positives in dbSNP can be conservatively estimated by resequencing DNA that has been collected from many individuals. SNPs that cannot be validated in such a manner are either rare SNPs that were not present in the sample or are false positives of the SNP discovery method. Recent resequencing studies validate 60–86% of the entries in dbSNP (Carlson et al. 2003; Reich et al. 2003). We anticipated a lower rate of false positives in the 8,408 SNPs that were used in this analysis because we removed error-prone categories of SNPs (such as those derived from ESTs or duplicated regions) from our data set. Despite this expected improvement, our initial analysis focused on the subset of 2,561 SNPs that had been validated by resequencing and were thus assumed to be free of errors (see Materials and Methods). This precaution was taken because the measurement of ESE disruption is particularly sensitive to artifacts (unpublished data).

**Figure 2:**
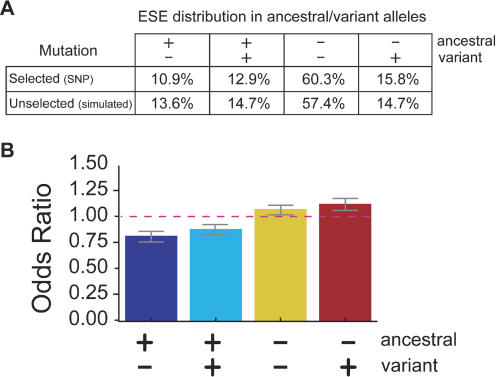
Analysis of the Effects of SNPs and Unselected Mutations on Predicted ESEs (A) The percentages of the four prediction outcomes. ESE disruption (+ −), ESE alteration (+ +), ESE neutrality (− −), and ESE creation (− +) changes are listed for the set of 2,561 validated SNPs (selected) and for the set of 100,000 simulated (unselected) mutations. (B) Synonymous and nonsynonymous mutations were analyzed separately and then compared using the MH test for homogeneity. All outcomes passed the MH test for homogeneity (H_0_:Outcome_synon_ ≈ Outcome_nonsynon_; *p* < 0.05) and could, therefore, be combined into a summary OR (weighted combination of the ORs measured in the synonymous and nonsynonymous sets). The height of each bar can be interpreted as the odds that the listed outcome will occur in the evolutionarily selected set of mutations (SNPs) relative to the odds that the same outcome will occur in the unselected (simulated mutation) set. Error bars extend one standard deviation on either side of the calculated value.

The annotation of RESCUE-ESE hexamers in a biallelic SNP results in one of four possible outcomes: no ESE hexamers in either allele (ESE neutrality, − −), one or more ESE hexamers only in the ancestral allele (ESE disruption, + −), one or more ESE hexamers only in the variant allele (ESE creation, − +), or one or more ESE hexamers in both alleles (ESE alteration, + +). The latter category is referred to as ESE alteration because the sets of RESCUE-ESE hexamers in the ancestral and variant alleles are, of course, different, and therefore may not necessarily be recognized with the same affinity by the same *trans*-factor(s). The relative frequencies of these four outcomes are listed in Figure 2A in the row labeled “Selected (SNP).” Since the 238 RESCUE-ESE hexamers represent only a small fraction (approximately 6%) of the 4,096 possible hexanucleotides, it was not surprising that a large majority of SNPs fell into the ESE-neutral category. To determine whether the rate of ESE disruption in SNPs was higher or lower than what would be expected from unselected mutations, we performed a Monte Carlo (random) simulation of point mutations in human exons.

### Reduced Frequency of ESE Disruption in SNPs versus Unselected Changes

Mutations were simulated at random in human exons using nucleotide substitution frequencies that reflect the mutational biases observed in unselected regions of the genome with similar nucleotide composition. In vertebrates, the greatest biases in nucleotide-to-nucleotide substitution frequencies are related to the hypermutability of C residues (Duncan and Miller 1980) and the higher rate of transitions (C ↔ T, A ↔ G) relative to transversions (purine ↔ pyrimidine). Rates for different base changes have been estimated from analysis of aligned sequences which are assumed to be under no selective pressure. For example, transitions make up 67–70% of all substitutions in human pseudogenes and 65.5% of all substitutions in genomic repeat sequences (Graur and Li 2000; Hardison et al. 2003; Zhang and Gerstein 2003).

Using the substitution frequencies derived from a recent large-scale study of nucleotide substitution patterns in processed pseudogenes (Zhang and Gerstein 2003), mutations were simulated in the set of approximately 121,000 GENOA-annotated internal human exons and the effects on predicted ESEs were analyzed as described above for SNPs. The simulation captured the influence of nearest-neighbor bases on the pattern and rate of nucleotide substitution. These nearest-neighbor effects are particularly pronounced for CpG dinucleotides, where an elevated C-to-T mutation rate is observed as an indirect consequence of cytosine hypermethylation. Comparing the results of this simulation to those observed for SNPs (listed as “Selected (SNPs)” in Figure 2A), the most striking difference was observed for the category of ESE disruption: 13.6% of simulated (unselected) mutations caused ESE disruption compared to only 10.9% of SNP mutations. This difference implies a significant selective disadvantage for mutations that disrupt RESCUE-ESE hexamers.

To assess the degree of selection on ESEs, one standard measure is the relative risk (RR), defined in this instance as the ratio of the frequency of ESE disruption in SNPs to the frequency of ESE disruption for unselected mutations (e.g., RR = 10.9%/13.6% = 0.80, using the pooled data from Figure 2A). In this instance, we preferred to use the slightly more complex odds ratio (OR) measure to quantify this effect (defined in Materials and Methods) because of its better statistical properties (Pagano and Gauvreau 2000).

As expected, the SNP dataset was greatly enriched for synonymous variation relative to the simulated mutation dataset. There is a 1.3:1 ratio of synonymous:nonsynonymous changes in the SNP dataset compared to a 0.5:1 ratio of synonymous:nonsynonymous changes in the simulated dataset. This difference, which has been observed many times, suggests that more than 60% of mutations that change the amino acid sequence are eliminated by natural selection (Graur and Li 2000). In order to account for the potentially confounding effect of selection occurring at the protein level, SNPs and simulated mutations were divided into synonymous and nonsynonymous groups and analyzed separately. After controlling for the higher frequency of synonymous mutations in dbSNP, the selective pressure to avoid disrupting ESEs was approximately equal for the synonymous and nonsynonymous classes of mutations (the Mantel–Haenzel [MH] test of homogeneity indicated no significant differences in the magnitude of the effect across all comparisons; χ^2^ < 0.5). This observation confirms our previous result and alleviates the concern that the analysis might have been confounded by the effects of selection acting at the protein level. The summary OR (the weighted combination of the separate synonymous and nonsynonymous analysis) for ESE disruption was 0.82 ± 0.05 (Figure 2B), which implies that natural selection has eliminated approximately 18% of arising point mutations that disrupt RESCUE-ESE hexamers (*p* < 0.001).

Base changes that alter one or more predicted ESE hexamers but result in creation of other RESCUE-ESE hexamers (ESE alteration) are also selected against, but to a somewhat lesser degree (Figure 2B, “+ +” category). This observation is not surprising given that these changes may alter the specific combination of SR proteins which interact with the exon and consequently alter ESE activity. In our previous study, we found that ESE alteration mutations would often cause an increase or decrease in enhancer activity, as determined in vivo using a splicing reporter construct (Fairbrother et al. 2002). SR proteins generally have distinct, though sometimes overlapping, RNA binding specificities and vary in their ability to activate splicing (Graveley et al. 1998; Liu et al. 1998). Therefore, some ESE alteration mutations that result in one SR protein replacing another SR protein may weaken the ESE and disrupt splicing. In addition, there are situations where the simultaneous binding of multiple activator proteins on a substrate is critical for correct processing of that pre-mRNA (Tian and Maniatis 1993). In such a case, it is unlikely that one ESE could be exchanged for another without deleterious consequences.

At first glance, the overrepresentation of some categories in this analysis may seem surprising. However, this is simply a consequence of the underrepresentation of disruption and alteration mutations in the SNP pool causing the remaining two categories of variation, ESE neutrality and ESE creation, to appear slightly overrepresented in SNPs relative to unselected mutations (Figure 2B). As mutations that result in a selective disadvantage are rapidly eliminated from the population, an increasing fraction of the mutations that persist as SNPs will be selectively neutral (Graur and Li 2000). In other words a neutral variant will, on average, be eliminated less rapidly than a disadvantageous variant and so the set of neutral variations will come to represent an increasing fraction of the total (diminishing) pool of variants. The analysis presented here divides SNPs into four categories based on ESE annotation. As variations from two of these categories (ESE disruption and ESE alteration) are shown to be preferentially eliminated by natural selection, the remaining two categories (ESE neutrality and ESE creation) will represent a larger fraction of a diminished total pool and, therefore, appear enriched (Figure 2B).

### Selective Pressure Is Strongest for ESEs Located near Splice Sites

**Figure 3:**
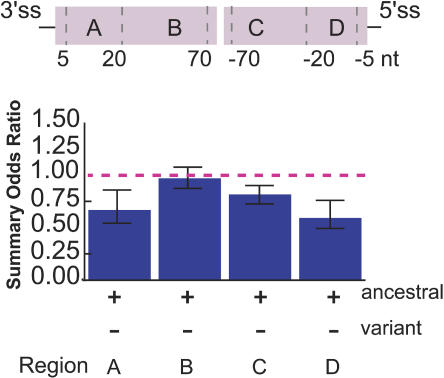
Selection against Disruption of Predicted ESEs in Different Exon Regions Summary ORs were calculated for mutations that disrupt RESCUE-ESEs as in Figure 2, for each of four regions spanning the length of a typical human internal exon. The heights of the blue bars represent the odds that an ESE will be disrupted by a mutation in the set of 2,561 validated SNPs (selected mutations) relative to the odds of disruption in the set of 100,000 simulated (unselected) mutations. Error bars extend one standard deviation on either side of the calculated value.

Experiments measuring the splicing activity of substrates with variations in the distance between a well-characterized ESE and the 3′ss have demonstrated a strong proximity effect, with ESE activity decreasing as the distance from the splice site increases (Lavigueur et al. 1993; Graveley et al. 1998). Although the closest ESEs tested in the distance studies were 70 nucleotides away from the splice sites, other studies have demonstrated that ESEs can function at much closer distances to splice sites (Nelson and Green 1988; Coulter et al. 1997). In order to test the generality of this distance effect, we quantitated the selective pressure on ESEs in distal and proximal windows at both the 3′ss and the 5′ss (Figure 3). Validated SNPs that fell within a particular exon region (e.g., the first 20 nucleotides of the 3′ss proximal window is defined as region A in Figure 3) were compared to unselected (simulated) mutations that fell in the same region, and summary ORs for ESE disruption were calculated for the four exon regions shown. Consistent with higher ESE activity for ESEs located near splice sites, we observed a pronounced increase in the conservation of RESCUE-ESE hexamers located within 20 bases of either the 5′ss or 3′ss (*p* < 0.05) relative to predicted ESEs located further from splice sites (Figure 3).

### SNP Analysis Identifies Conserved ESEs

Our observations that SNPs tend to avoid disrupting RESCUE-ESE hexamers (see Figure 2) and that the magnitude of this selection increases near splice sites (Figure 3) indicate that this set of hexamers represents a physiologically important collection of sequences across many human exons and genes. Although all RESCUE-ESE hexamers tested to date have ESE activity in cell culture assays, we have tested only a small fraction of the 238 individual hexamers, and presumably some members of this set may be false positives of the RESCUE-ESE method.

**Figure 4:**
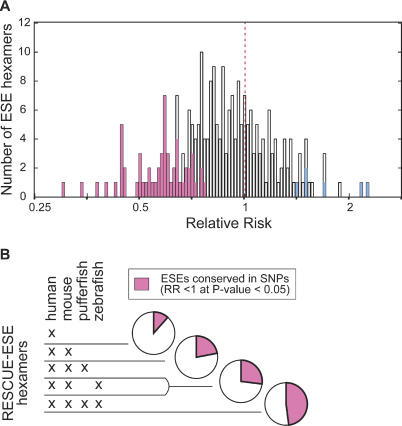
Measuring Selective Pressure on Each RESCUE-ESE Hexamer Any point mutation alters six overlapping hexamers, and so a database of 8,408 SNP mutations alters a total of approximately 50,000 hexamers in the wild-type (ancestral) allele. In considering all 238 RESCUE-ESE hexamers, the frequency of each ESE hexamer in the total set of ancestral alleles was recorded for the database of SNPs and simulated mutations (8,408 SNP mutations and 100,000 simulated mutations). The ESE frequency in the SNP set was divided by the ESE frequency in the simulated set to calculate the RR for each of the 238 hexamers. (A) The distribution of RR for all 238 ESE hexamers is plotted on a logarithmic scale. A resampling strategy was used to identify 57 ESE hexamers that were significantly conserved (pink bars have an RR less than 1; *p* < 0.05) and also six ESE hexamers that were not conserved (blue bars have an RR greater than 1; *p* < 0.05). (B) The output of RESCUE-ESE was compared for several vertebrate genomes (human, mouse, pufferfish, and zebrafish). The set of 238 human RESCUE-ESE hexamers was divided into nonoverlapping subsets based on their conservation in the RESCUE-ESE output generated from other vertebrates. The proportion of ESEs that were significantly conserved in the SNP analysis (as described above in [A]) were recorded for each subset of RESCUE-ESE hexamers and are represented as pink sectors in the pie chart.

In order to better define functional hexamers in the RESCUE-ESE set, we repeated the selected versus unselected comparisons for each hexamer individually using the larger set of 8,408 exonic SNPs described previously. For each RESCUE-ESE hexamer, we counted cases where a hexamer was interrupted by a mutation that has survived selection (SNP mutations) and compared this frequency to the value we would expect in the absence of selection (simulated mutations). For this analysis we used a simple RR measure, defined as the frequency with which a hexamer overlaps with SNPs relative to the frequency with which the same hexamer overlaps with simulated mutations. This ratio (frequency_SNP_/frequency_simulated_) was calculated for each hexamer and provided a means of assessing the selective pressure on each hexamer. The RR for a hexamer that was under no additional selective pressure would therefore be equal to 1.0, and a hexamer under increased selection would have an RR of less than 1. Consistent with our previous analysis (see Figure 2), the majority of RESCUE-ESE hexamers (162 hexamers) had an RR of less than 1 (Figure 4A), suggesting that many RESCUE-ESE hexamers are subject to purifying selection.

While the somewhat limited size of the currently available SNP databases limits our power to detect selection acting on individual hexamers, it was possible to detect a subset of hexamers that displayed a statistically significant level of conservation. A bootstrap sampling strategy identified a total of 57 hexamers with RRs of significantly less than 1 (Figure 4A, pink bars) compared to only six hexamers with RRs significantly greater than 1 (Figure 4A, blue bars). By comparison, testing a set of 238 arbitrary hexamers would be expected to yield approximately 12 significant hexamers in each of these categories at a *p* value cutoff of 0.05. Included within the set of 57 conserved ESEs are hexamers corresponding to the well-characterized “GAR” and AC-rich ESE classes (Coulter et al. 1997) and several other types of ESEs (see Supporting Information).

A comparison of RESCUE-ESE predictions for four different vertebrates (G. Yeo, S. Hoon, B. Venketesh, and C. B. B., unpublished data) revealed a strong correspondence between within-species conservation (SNP analysis) and cross-species conservation (Figure 4B). In other words, the ESE hexamers that appeared to be under the greatest selective pressure within the time frame of the SNP analysis (the last hundreds of thousands of years) were more likely to retain the characteristics used by the RESCUE approach to identify ESEs over the time frame of vertebrate speciation (the last tens to hundreds of millions of years). While only a minor fraction (11%) of the hexamers that appear exclusively in the human lineage were significantly conserved in the SNP analysis (RR < 1; *p* < 0.05), about half of the hexamers predicted to be ESEs in all four vertebrates examined (human, mouse, zebrafish, and pufferfish) were significantly conserved in the SNP analysis. In the future, with a larger SNP database and more genomes available, it should be possible to use these methods to analyze more individual hexamers for evidence of selective pressure.

As mentioned previously, the public SNP database used in this study contains a significant fraction of entries that could not be validated by resequencing. If these unvalidated entries in dbSNP were errors, they would not be expected to specifically avoid ESEs, or particular exon positions. Therefore, SNP artifacts are likely to reduce, rather than increase, the apparent significance of the biases in the nature and distribution of SNPs that we have observed.

Here we have shown, using polymorphism data, that RESCUE-ESE hexamers have been preferentially conserved in the recent evolutionary history of the human lineage and that the strength of this conservation increases with increasing proximity to the splice sites. These results imply that splicing imposes important constraints on the evolution of human exons.

As the size and quality of the SNP database is rapidly increasing, measures of selection deriving from SNP data are likely to become increasingly useful for evaluating the function of short, degenerate sequence elements like ESEs. For example, it should be possible to use the VERIFY method to analyze selection on motifs that are postulated to control transcription, polyadenylation, messenger RNA (mRNA) stability, or translation. The current build of dbSNP contains 360,000 SNPs that are located in a “locus region” within 2 kb upstream of the transcript start site or 500 bp downstream of the polyadenylation site (Sherry et al. 2001). There are also more than 500,000 SNPs localized to the untranslated regions of mRNAs where elements that modulate translation, polyadenylation, and mRNA stability are thought to be located. The analysis presented here used 8,400 SNPs to study selection on ESE hexamers. Making the simplifying assumptions that ESE hexamer frequencies are both uniform and slightly elevated in exons and are mutated without bias, we can roughly estimate the statistical power of the VERIFY method to detect selection (see Materials and Methods). For example, the set of ESE hexamers, as a whole, is under selective pressure (OR = 0.82), but this selective pressure will vary according to hexamer, with some hexamers being highly conserved and others less so (Figure 4). Our ability to detect conservation at the level of the individual hexamers depends upon (1) the degree of hexamer conservation (large differences are easier to detect than small differences) and (2) the number of times a SNP interrupts an ESE hexamer (larger data size increases our confidence in individual measurements and, therefore, increases our ability to measure differences). The VERIFY method could detect about 70% of strongly conserved hexamers (OR > 0.5) in the set of 238 ESE hexamers using 8,400 SNPs. A similar type of analysis performed with 500,000 SNPs would not only detect almost all the strongly conserved motifs but would be able to detect motifs under a weaker degree of selection (OR > 0.77) with a similar power (approximately 70%). As an alternative to using polymorphisms within a species, VERIFY could also utilize variations between closely related species to study selection on gene control elements.

In addition, this work identifies two new categories of mutations that are under selective pressure: mutations that disrupt or alter RESCUE-ESE hexamers. This increased selective pressure presumably reflects the increased likelihood that such variations will alter splicing and, therefore, gene activity. Several cases of polymorphisms which result in allele-specific differences in splicing have been reported (Betticher et al. 1995; Stallings-Mann et al. 1996; Stanton et al. 2003). Here, we identify features such as proximity to splice sites and ESE disruption and alteration that should prove useful in discovering additional polymorphisms that affect splicing. As splicing mutations constitute at least 14% of disease-causing mutations (Stenson et al. 2003), polymorphisms that affect splicing would be good candidates for association studies intended to identify genetic contributors to quantitative traits or diseases. An additional benefit of using variations that are predicted to result in altered splicing relates to the feasibility of validating an RNA phenotype. While variations predicted to alter protein activity may require gene-specific activity assays or antibodies, RNA phenotypes such as exon skipping can be readily screened by RT-PCR, thus enabling large scale phenotyping of genotyped cell lines.

## Materials and Methods

### 

#### SNP data

Build 112 of dbSNP was downloaded from the NCBI ftp server (ftp.ncbi.nih.gov) and parsed from the XML format, which integrates data (e.g., annotation, sequence, transcript) from several sources (Sherry et al. 2001). A current description of the database structure is available online (http://ncbi.nih.gov/). To limit the contribution of erroneous SNPs, several filters were applied to produce the set of SNPs used in this analysis. First, SNPs located within a coding region in the assembly annotation were required to align to an internal exon in the GENOA exon dataset. GENOA is a genome annotation script that annotates exons by spliced alignment of mRNA/cDNA sequence to an assembled genome, applying a number of checks on the quality of the resulting alignments (see below). SNPs that mapped to multiple genomic regions, to known repetitive elements, to regions where the reading frame differed in the genomic/transcript alignment, or to an entry that had been contributed in the context of spliced transcripts (e.g., through comparison of ESTs) were excluded. We also removed entries where both versions of a SNP contained a match to either the human exon database or the chimpanzee *(Pan troglodytes)* genome (ftp://ftp.ncbi.nih.gov/pub/TraceDB/pan_troglodytes/). From the resulting pool of reference SNPs, entries associated with genotype data (population frequency) were used to build a validated SNP set. Unless otherwise indicated, SNPs were aligned to other sequences only when a perfect (33/33) base match was obtained using BLAST (Altschul et al. 1997) with one or the other of the polymorphic alleles.

#### Exon data

Datasets of internal human exons were generated by spliced alignment of cDNA sequences from GenBank (release no. 134) to the assembled, masked human genome sequence (GoldenPath assembly HG13, http://genome.ucsc.edu) using the genome annotation script GENOA (D. H., Lee P. Lim, R.-F. Yeh, U. Ohler, and CBB, unpublished data). The exon/intron structures were inferred from at least two cDNA/genomic alignments, and the reading frame was determined from the CDS annotation of the GenBank cDNAs. As RESCUE-ESE hexamers were identified in constitutively spliced exons and alternative splicing sometimes results in ambiguous splice site positions, we confined our analysis to exons which showed no (cDNA) evidence of alternative splicing. Therefore, exons present in some, but not all, cDNA alignments overlapping a genomic region were excluded. In addition, it was required that each exon (1) be flanked by introns whose ends matched the consensus terminal dinucleotides of U2-type or U12-type introns (GT-AG, GC-AG, or AT-AC) and (2) be in an open reading frame that spanned at least three exons (the exon being considered and at least one flanking exon on either side).

#### Simulation of exon mutations

A Monte Carlo simulation was used to estimate background frequencies, in humans, of ESE-disrupting, ESE-creating, ESE-neutral, and ESE-altering mutations in internal exons. A PERL script simulated point mutations in sequences during multiple passes through the GENOA exon dataset. For each exon considered, the script utilized a set of randomly generated numbers to dictate the following sequence of decisions: (1) whether a mutation would occur in an exon, (2) the position where a mutation would occur, and (3) the identity of the variant allele. Mutations were simulated in present-day internal coding exons with a probability of occurrence proportional to the length of the exon and no a priori strand or position bias. The output mutations were stored in reference SNP format and annotated with predicted ESEs as described in the text.

The mutation probabilities and the pattern of substitution are functions of the input sequence (i.e., the base to be mutated and the 3′ neighboring base). The values of all 12 nucleotide-to-nucleotide substitution probabilities used here were considered for all four possible 3′ neighboring-base contexts. The distribution of dinucleotide substitution probabilities (48 possible) used in this work was derived from a large study of mutation in ribosomal protein pseudogenes (all substitution probabilities were extracted from Figure 2 except those for CpG, which were obtained from Figure 1 using the assumption that both transversion values, i.e., CpG to ApG and CpG to GpG, were of equal probability [Zhang and Gerstein 2003]). These probabilities reflect the context effect of the nucleotide 3′ of the substituted base averaged over all four possible 5′ contexts. If neighboring-nucleotide effects are not independent and the relevant context encompasses more sequence than is being considered in the simulation, this may result in a slight effect on the substitution pattern generated by the simulation. The dominant well-known contextual influences, such as those seen in the CpG dinucleotide, are captured in the simulation. Total genomic sequence was used to derive the substitution rates and simulate mutations. Features that could potentially influence the mutation process, such as the extent of CpG methylation, regional GC content, or recombination rates, could not be explicitly incorporated into the simulation. However, the analysis and simulation excluded first exons (reducing the contribution of unmethylated CpG in CpG islands), and these other variables affected the result of this analysis only to the degree that they altered the substitution pattern, as distinct from the overall mutation rate.

#### Analysis

Mutations were annotated in terms of their effect on overlapping RESCUE-ESE hexamers as ESE disruption (+ −), ESE creation (− +), ESE alteration (+ +), or ESE neutrality (− −). In the annotations, the first position refers to the wild-type or ancestral allele, where “+” indicates one or more ESE hexamers and “−” indicates no ESE hexamers. The ESE alteration category can include mutations in which a net gain or a net loss of ESE hexamers is annotated. All annotations used the set of 238 RESCUE-ESE hexamers described previously (Fairbrother et al. 2002).

ORs (rather than RRs) were used to quantify the difference between the set of experimentally validated SNPs and simulated mutations in Figures 2 and 3. Briefly, the relative OR is defined here as



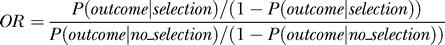



where P(outcome|selection) represents the probability that SNP (a mutation under selection) will have a particular ESE annotation outcome (+ +, + −, − −, or − +). The extent of selection was measured separately for synonymous and nonsynonymous mutations and standard methods were used to calculate 95% confidence intervals (Pagano and Gauvreau 2000). The MH test was used to determine whether the degree of selection was independent of synonymy.

Experimentally validated SNPs that were located in the first or last 70 nucleotides of an exon were evaluated for ESE disruption as a function of position. As no restrictions were placed on exon size, it was possible for SNPs in small exons to be placed in multiple categories (as was the case for approximately 20% of all SNPs). ORs were used to measure the extent of selection, and significance was assessed at an α level of 0.05 (one-tailed) for ESE disruption mutations in the four different regions of an exon.

Bootstrap sampling was used to determine hexamers that were significantly avoided by SNPs. RR was used to compare the selection on individual hexamers over the course of 5,000 trials. For each trial, 8,408 SNPs were sampled with replacement from the set of 8,408 SNPs described in the text. The frequency of cases where an ESE coincided with an SNP was calculated for each of the 238 RESCUE-ESE hexamers and divided by the expected frequency for that hexamer (determined through simulation). This ratio of frequencies was used to estimate RR for each hexamer. Dividing the instances where RR was greater than 1 by the number of trials (5,000) provided a bootstrap *p* value for each of the 238 hexamers.

The interspecies comparisons considered the output of RESCUE-ESE on four vertebrate genomes: human, mouse, zebrafish, and pufferfish. The set of human hexamers was divided into subsets according to the degree of conservation across vertebrates in the following manner: Hexamers that were only present in humans defined set 1; hexamers present in human and mouse, set 2; hexamers present in human, mouse, and one of the fish species, set 3; hexamers present in human, mouse, zebrafish, and pufferfish, set 4. The degree of overlap between these hexamer sets and the RESCUE-ESE hexamers with an RR of significantly less than 1 was recorded and displayed in Figure 4B.

Power calculations for single hexamer analysis were performed as described previously (Pagano and Gauvreau 2000; method, pages 243–246; estimation of standard error as a function of sample size, page 355). For the purposes of the power calculation the mutations were assumed to be unbiased and the ESE hexamers were assumed to have a slightly elevated frequency (1.5/4,096 = 3.7 × 10^−4^ rather than 2.4 × 10^−4^), and so a particular ESE would be expected to be interrupted by a SNP at any one of six positions with a frequency of 6 × 3.7 × 10^−4^ = 2.2 × 10^−3^. This probability was used to estimate the number of variations that would interrupt (disrupt or alter) an ESE hexamer with and without selection for the different SNP database sizes and degrees of selection (ORs) described in the text.

## Supporting Information

### URLs

http://genes.mit.edu/burgelab/rescue-ese/. An online tool to annotate RESCUE-ESE hexamers in exons.

http://genes.mit.edu/burgelab/Supplementary/fairbrother04/. Contains exon, SNP, RR, and ancestral allele databases used and/or generated in this study.

## Abbreviations

3′ss: 3′ splice site
5′ss: 5′ splice site
dbSNP: public SNP database
ESE: exonic splicing enhancer
EST: expressed sequence tag
MH test: Mantel–Haenzel test
mRNA: messenger RNA
OR: odds ratio
RESCUE: relative enhancer and silencer classification by unanimous enrichment
RR: relative risk
PERL: Practical Extraction and Report Language
SNP: single nucleotide polymorphism
VERIFY: variant elimination reinforces functionality
